# Finding missing TB cases in Northern Nigeria

**DOI:** 10.5588/pha.24.0045

**Published:** 2025-03-01

**Authors:** S.A. Omotayo, O. Chukwuogo, C. Ogbudebe, D. Egbule, P. Opara, T. Bot, E. Chukwu, P. Nwadike, I. Gordon, C. Ezekhaigbe, A. Yakubu, B. Odume

**Affiliations:** ^1^ KNCV Nigeria, Abuja, Nigeria;; ^2^ RedAid, Enugu State, Nigeria;; ^3^ Initiative for Prevention and Control of Diseases, Nasarawa State, Nigeria;; ^4^ Center for Bioethics and Research, Ibadan, Oyo State, Nigeria.

**Keywords:** TB, intensified case finding, Northern Nigeria, public hospital, TB LON 64

## Abstract

**SETTING:**

Despite recent progress in TB notification rates, 6.2% of the 3.1 million ‘missing’ people with TB globally are from Nigeria. Identifying these ‘missing’ cases will improve TB control efforts in Nigeria.

**OBJECTIVE:**

This paper aims to describe the outcome of an intensified TB case-finding strategy in northern Nigeria.

**DESIGN:**

An intensified TB case-finding strategy was implemented in four states in northern Nigeria from October 2021 to September 2022. Trained ad-hoc staff screened hospital attendees and linked identified persons with presumptive TB to diagnosis using a hub and spoke approach. People with confirmed TB were linked to treatment. Contributions of the strategy to the national TB notification rates for each state were assessed.

**RESULTS:**

A total of 1.17 million individuals were screened for TB across the four project States. 64,079 people with presumptive TB were identified, of which 10.1% were diagnosed with TB and 97% of those diagnosed were placed on treatment. Averagely, 33.3% of the TB cases notified from each state were contributions from the hospital-based Intensified TB case-finding intervention.

**CONCLUSION:**

Facility-based intensified TB case-finding results in significant improvement in TB notification rates and a good strategy to improve the identification of missing TB cases in Nigeria.

Despite a 63% reduction in age-standardised disability-adjusted life years (DALYs) between 1990 and 2019,^1^ TB remains one of the top causes of death worldwide from infectious diseases.^2^ Partly due to the lingering effects of COVID-19, 7.5 million new people with TB were diagnosed globally in 2022, compared to 7.1 million and 5.8 million cases in 2019 and 2020, respectively. There are still 3.1 million missing TB cases globally.^2^ With the present trend, the world is not on track to meet the Stop TB Partnership and the WHO’s milestones for ending TB by 2030^3^ and 2035, respectively.^4^

Nigeria has the highest burden of TB in Africa and is responsible for 4.5% of the global burden of TB. Although it recorded a sustained increase in TB notification rate throughout the COVID-19 pandemic, Nigeria still accounts for 6.2% of the world’s 3.1 million ‘missing’ people with TB (new or relapse cases notified that fall short of the best estimated TB incidence).^2^ This is because of various factors, including poor access to health services, missed people with presumptive TB in outpatient departments (OPDs), and failure of laboratory testing of people with presumptive TB.^5^ Ensuring that Nigeria can increasingly find these ‘missing’ people with TB can significantly improve the country’s TB control efforts.^6^

Facility-based intensified case-finding (ICF) for TB (also called active TB case-finding) is a cost-effective, high-impact approach that can help identify up to one-third of missing people with TB.^7^ In Nigeria, facility-based ICF is one of the key government-approved and WHO-recommended strategies for TB control.^8^ Despite the policy supporting TB ICF, the number of missing people with TB remains alarmingly high in Nigeria. This may be because active TB screening was mostly limited to OPDs and due to other health and non-health-related barriers to effective TB ICF. These include limited experience and knowledge of healthcare workers in screening and detecting persons with active TB, limited availability and high workload of healthcare workers involved in TB management in the facilities, and inadequate resources for diagnosis and management of persons with TB among others.^9^ We designed and implemented a facility-based TB ICF strategy as part of the USAID-funded TB Local Organizations Network (LON) Regions 1 & 2 Project (TB LON 1&2)^10^ to address these challenges. In this paper, we report the outcome of the TB ICF strategy and its contribution to the total TB case notification from public health facilities in four northern states.

## Study population, design, and methods

KNCV Nigeria implemented the TB LON 1&2 project in collaboration with the private sector, health and allied professional organisations, faith-based institutions, communities, and civil society. It aims to address health and non-health-related barriers to significantly increase the number of persons with TB detected, treated, and notified within the project’s 5-year grant implementation period from 2020 to 2025. Generally, the project is implemented across 14 states in Nigeria comprising Akwa Ibom, Anambra, Bauchi, Benue, Cross River, Delta, Imo, Kaduna, Kano, Katsina, Nasarawa, Plateau, Rivers and Taraba. This study reports on the outcome of the TB ICF strategy in public health facilities in Benue, Nasarawa, Plateau, and Taraba states. These are among the top 10 states with the highest burden of TB in Nigeria after Sokoto^1^ (Figure).

**FIGURE.**
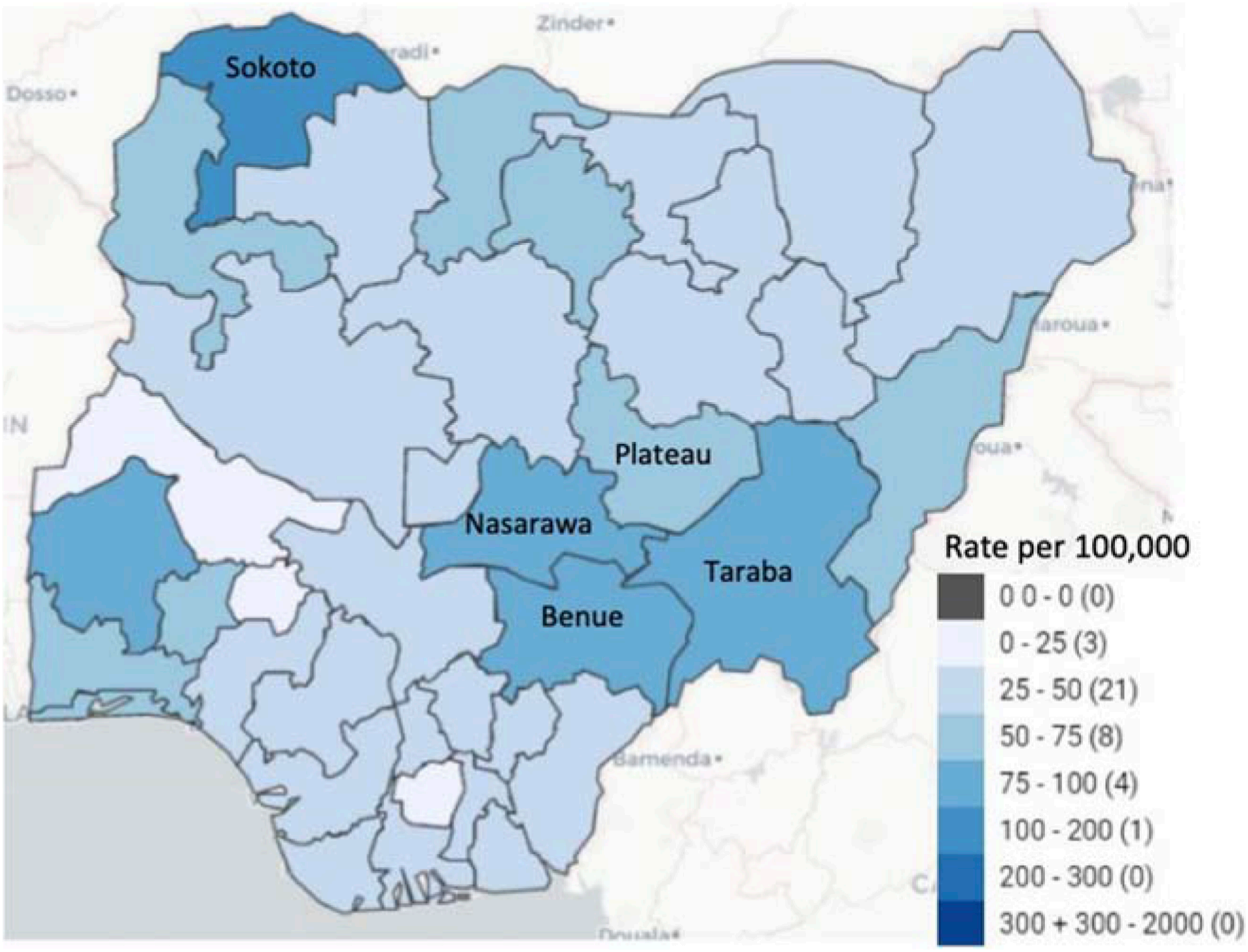
TB notification rates in Nigeria by state, 2018. Source: National Tuberculosis, Leprosy and Buruli Ulcer Control Programme. Nigeria Tuberculosis Epidemic. Abuja, Nigeria: NTBLCP, 2020.

To improve on the predominantly OPD-based TB ICF that is the practice in public facilities and to address the health workforce and other TB service-related operational challenges, the following strategies were implemented: 1) expansion of the service delivery points at which patients are screened; 2) recruitment and training of ad-hoc staff on active TB screening, and diagnostic-referral; 3) active follow-up and linkage of confirmed TB cases to DOTS treatment centres nearest to them for treatment. The TBLON Project is implemented in the roll-in, roll-on and roll-out phases. The roll-in phase, from March 2020–September 2021, involved recruitment and training of project and ad-hoc staff and onboarding of facilities selected for the project. The TB ICF strategy at the selected facilities fully commenced during the roll-on phase, starting in October 2021.

During the roll-in phase, at each of the 257 public health facilities (8 tertiary, 48 secondary, and 201 primary health facilities), ad-hoc staff were recruited and assigned to the available service delivery points (SDPs). They screen and provide appropriate support to persons with presumed TB. All ad-hoc staff were trained with certificated competency-based tests continuously. The training focused on the epidemiology of TB, identification of persons with presumptive TB, specimen collection, infection control, and linking patients to treatment. They were also trained in using reporting tools to record data and capture data electronically. At a minimum, ad-hoc staff had completed high school and possessed the ability to read, write, and communicate effectively in both English and the predominant language of their respective states.

For the roll-on phase, active TB screening was initiated at the designated SDPs for all patients visiting the facilities and their accompanying relations. The SDPs at which patients were screened include the traditional general OPD, antiretroviral treatment (ART) clinics, accident and emergency, antenatal care, child welfare, immunisation, and diabetes clinics. Others include medical wards (male and female), medical OPD, and paediatric OPD. TB screening was done using the WHO 4-symptom screen approach.^11^

A hub-spoke approach was used to refer samples of people with presumptive TB for diagnostic confirmation and for linking those with confirmed TB to care. A hub has at least one molecular WHO-recommended rapid diagnostic platform. About 98% of the hub sites are in tertiary and secondary health facilities with designated units for DOTS service. On the other hand, spokes are functional primary healthcare facilities offering health services and attending to at least 10 clients daily. People with presumptive TB from the spokes were actively linked to a suitable hub based on proximity to the patient’s location or choice for further evaluation. For this purpose, ad hoc staff designated as linkage coordinators collect the sputum from the presumptive TB persons and send it to a suitable hub following a standard protocol established for the program. S/he ensures the timely receipt and processing of the samples at the diagnostic testing sites and also follows up to ensure that results are available within 48 hours of submission of the samples for testing.

Evaluation to confirm active TB was done using available molecular diagnostic tools – Xpert^®^ MTB/RIF assay (Cepheid, Sunnyvale, CA, USA), loop-mediated isothermal amplification (TB-LAMP) assay, or Truenat^TM^ (Molbio Diagnostics, Verna, India) and microscopy assay for those that can and by chest X-ray evaluation for those who can not produce sputum. In collaboration with local government TB supervisors, persons identified with active TB were immediately placed on treatment and properly documented in the national registers.

## Data collection and management

Data for each patient was collected using the COMMCARE app. This is a real-time screening and reporting app, used in the TBLON project to capture data on people with presumptive TB, their linkage from spoke to hub sites, and follow-up to capture people with active TB and their placement on treatment. The app also synchronises the demographic details (de-identified) of people with confirmed TB through the backend to the cloud for geospatial hotspot mapping and EWORS (early warning TB outbreak recognition system) for improved programming.

Periodic meetings were conducted weekly to review performance and ensure data quality at the site level and monthly at the state level. TB control cascade indicators were monitored to ensure no patients were missed. Additionally, spatial data were used to prioritise sites with high yield for special attention.

## Data analysis

We compared the number of those with active TB identified through our TB ICF strategy with those identified through routine state TB programmes to assess the contribution of our intervention to the total TB notification rates in each of the four states. All data were presented descriptively.

## Ethical consideration

The study was determined to be a non-research programme evaluation. As it required no direct contact with human subjects (no interview or sample collection), and only de-identified pooled program data that formed part of the standard of care were used, informed consent was not required.

## RESULTS/IMPACT:

A total of 237 staff were recruited and trained for the intervention. Of the 237 staff, 106 (45%) were male and 131 (55%) were female. The age of the staff ranges from 22 to 47 years, and the average number of staff deployed per site is shown in Table 1.

**TABLE 1.** Distribution and demographic information of staff recruited, trained and deployed at sites to implement the interventions.

State	Number of sites (a)	Number of staff recruited and trained				Average number of staff recruited and trained/site (a/b)
		Sex		Total (b)	Age range years	
		Male	Female			
Benue	70	21	44	65	24–47	1
Nasarawa	68	38	24	62	25–44	1
Plateau	59	14	44	58	22–35	1
Taraba	60	33	19	52	25–35	1
Total	257	106	131	237	22–47	1

Table 2 shows the overall result of the TB ICF cascade for all four states from October 2021 to September 2022. The TB ICF was implemented in respectively 91%, 77%, 71%, and 69% of the local government areas (LGAs; equivalent to districts) in Benue, Nasarawa, Plateau, and Taraba. Overall, 1.17 million individuals were screened for TB across all four states, with a 5% average presumptive TB yield. Of the 64,079 people with presumptive TB identified, 6,456 (yield = 10.1%) were diagnosed with TB, and on average, 97% of them were put on an appropriate TB treatment regimen. TB presumptive yield, diagnostic testing yield, and treatment enrolment rates were comparable across all the states. Cumulatively, the mean number needed to screen (NNS) to identify a person with presumptive TB and the number needed to test (NNT) to diagnose a person with confirmed TB were respectively 185 (95% confidence interval [CI] 146–224) and 10 (95% CI 9–11).

**TABLE 2.** TB facility-based intensified case-finding cascade in four states, Northern Nigeria, October 2021–September 2022.

	Total LGAs	LGAs with TB LON	Total number screened	Presumptive TB cases and yield	Presumptive cases evaluated for TB	Confirmed TB cases and TB yield	Confirmed TB cases on treatment: enrolment rate	NNS	NNT
State	*n*	*n* (%)	*n*	*n* (%)	*n*	*n* (%)	*n* (%)		
Benue	23	21 (91.3)	333,307	19031 (5.7)	18,996	2120 (11.2)	2069 (97.6)	157	9
Nasarawa	13	10 (76.9)	293,319	17385 (5.9)	17,384	1549 (8.9)	1510 (97.5)	189	11
Plateau	17	12 (70.6)	295,349	13067 (4.4)	13,059	1369 (10.5)	1320 (96.4)	216	10
Taraba	16	11 (68.8)	250,954	14596 (5.8)	14,595	1418 (9.7)	1335 (94.1)	177	10
Total	69	54 (78.3)	1,172,929	64079 (5.5)	64,034	6456 (10.1)	6234 (96.6)	185	10

LGAs = local government areas; NNS = number needed to screen; NNT = number needed to test.

The age-disaggregated TB ICF cascade is presented in Table 3. Persons aged 25–34 and 35–44 years were screened the most frequently, respectively 253,745 (21.6%) and 200,626 (17.1%). These age groups also had the highest proportion of persons with presumptive TB and confirmed TB. Children aged 0–4 years and 5–14 years had the least proportion of persons with presumptive and confirmed TB compared to other age groups.

**TABLE 3.** Age- and sex-disaggregated TB LON public-ICF TB cascade data, October 2021–September 2022.

State	Sex			Age, years								
	Male	Female	Both sexes	0–4	5–14	15–24	25–34	35–44	45–54	55–64	≥65	Total (all ages)
Clients screened for TB												
Benue	134,262	199,045	333,307	41,169	25,968	50,351	66,285	57,019	41,777	28,324	22,414	333,307
Nasarawa	110,776	182,543	293,319	28,327	28,956	55,250	70,088	47,368	30,558	19,204	13,568	293,319
Plateau	115,160	180,189	295,349	30,394	27,817	46,145	59,344	51,739	36,521	24,057	19,332	295,349
Taraba	110,486	140,468	250,954	17,428	21,661	41,127	58,028	44,500	32,043	21,740	14,427	250,954
Total, *n* (%)	470,684 (40.1)	702,245 (59.9)	1,172,929 (100)	117,318 (10)	104,402 (8.9)	192,873 (16.4)	253,745 (21.6)	200,626 (17.1)	140,899 (12)	93,325 (8.0)	69,741 (5.9)	1,172,929 (100)
Clients with presumptive TB												
Benue	8,852	10,179	19,031	499	1,184	2,261	3,636	4,028	3,289	2,220	1,914	19,031
Nasarawa	7,475	9,910	17,385	835	1,299	2,721	4,398	3,571	2,324	1,234	1,003	17,385
Plateau	6,127	6,940	13,067	618	1,084	1,785	2,548	2,558	1,945	1,159	1,370	13,067
Taraba	7,238	7,358	14,596	422	913	2,046	3,278	2,932	2,347	1,565	1,093	14,596
Total, *n* (%)	29,692 (46.3)	34,387 (53.7)	64,079 (100)	2,374 (3.7)	4,480 (7.0)	8,813 (13.8)	13,860 (21.6)	13,089 (20.4)	9,905 (15.5)	6,178 (9.6)	5,380 (8.4)	64,079 (100)
Presumptive cases evaluated for active TB												
Benue	8,835	10,161	18,996	499	1,181	2,255	3,630	4,024	3,281	2,216	1,910	18,996
Nasarawa	7,475	9,909	17,384	835	1,299	2,720	4,398	3,571	2,324	1,234	1,003	17,384
Plateau	6,126	6,933	13,059	618	1,086	1,784	2,540	2,555	1,945	1,160	1,371	13,059
Taraba	7,238	7,357	14,595	422	913	2,049	3,275	2,930	2,347	1,566	1,093	14,595
Total, *n* (%)	29,674 (46.3)	34,360 (53.7)	64,034 (100)	2,374 (3.7)	4,479 (7.0)	8,808 (13.8)	13,843 (21.6)	13,080 (20.4)	9,897 (15.5)	6,176 (9.6)	5,377 (8.4)	64,034 (100)
Clients with active TB												
Benue	1,357	763	2,120	28	48	187	417	525	437	253	225	2,120
Nasarawa	943	606	1,549	71	83	224	440	331	215	111	74	1,549
Plateau	872	497	1,369	13	47	159	332	342	229	101	146	1,369
Taraba	871	547	1,418	20	49	171	379	323	222	118	136	1,418
Total, *n* (%)	4,043 (62.6)	2,413 (37.4)	6,456 (100)	132 (2.0)	227 (3.5)	741 (11.5)	1,568 (24.3)	1,521 (23.6)	1,103 (17.1)	583 (9.0)	581 (9.0)	6,456 (100)
Clients with active TB on Treatment												
Benue	1,319	750	2,069	28	46	183	407	511	429	249	216	2,069
Nasarawa	919	591	1,510	71	80	217	436	318	215	105	68	1,510
Plateau	843	477	1,320	12	46	157	317	331	218	99	140	1,320
Taraba	821	514	1,335	19	47	165	359	306	208	109	122	1,335
Total, *n* (%)	3,902 (62.6)	2,332 (37.4)	6,234 (100)	130 (2.1)	219 (3.5)	722 (11.6)	1,519 (24.4)	1,466 (23.5)	1,070 (17.2)	562 (9.0)	546 (8.8)	6,234 (100)

ICF = intensified case-finding; TB LON = TB Local Organizations Network.

The sex-disaggregated results of the TB ICF initiative are also shown in Table 3. Overall, more women were screened than men (60% versus 40%, respectively). Despite identifying more women with presumptive TB than men (53.7% versus 46.3%, respectively), cumulatively, fewer women had confirmed TB than men (62.6% versus 37.4%, respectively), and in each of the states.

Table 4 shows the quarterly contribution of our TB ICF initiative to the total TB notifications from our project states. Our project’s contribution to the total state TB notifications was highest in Plateau and Benue with respectively 38% and 36% contributions. Within each state, the number of TB cases notified through the TBLON ICF project to the National TB program in each of the quarters remained broadly consistent.

**TABLE 4.** Case identification comparison in TB LON ICF versus non-ICF facilities.

State	Period	ICF public TBLON Project notification	Total state TB notification	Contribution to total state TB notification %
Benue	Oct–Dec 2021	554	1,384	40.0
	Jan–Mar 2022	527	1,434	36.8
	Apr–Jun 2022	465	1,328	35.0
	Jul–Sep 2022	574	1,708	33.6
Average		530	1,464	36.4
Nasarawa	Oct–Dec 2021	366	1,160	31.6
	Jan–Mar 2022	372	1,307	28.5
	Apr–Jun 2022	423	1,416	29.9
	Jul–Sep 2022	388	1,157	33.5
Average		387	1,260	30.9
Plateau	Oct–Dec 2021	316	847	37.3
	Jan–Mar 2022	328	881	37.2
	Apr–Jun 2022	370	963	38.4
	Jul–Sep 2022	355	914	38.8
Average		342	901	38.0
Taraba	Oct–Dec 2021	394	1,199	32.9
	Jan–Mar 2022	290	1,218	23.8
	Apr–Jun 2022	357	1,227	29.1
	Jul–Sep 2022	377	1,439	26.2
Average		355	1,271	28.0
Cumulative average		403.5	1223.9	33.3%

LON = Local Organizations Network; ICF = intensified case-finding.

## DISCUSSION

The ICF intervention implemented in Benue, Nasarawa, Plateau and Taraba States resulted in the identification of 6,456 TB cases – a 10.1% diagnostic yield, and a 97% treatment linkage rate. The ICF intervention contributed to 33% of the TB cases notified to the national TB program across the four states. These results can be attributed to the expansion of services facilitated by interventions aimed at strengthening TB ICF in health facilities across the four states under the TBLON project. Studies in other West African countries, such as Tanzania and Kenya, have reported similar improvements in national TB notifications from ICF interventions.^12^ Studies have also reported efficient TB case identification in ART clinics through TB-HIV collaborative activities.^13^

Additionally, our innovation in optimising linkage (support for sputum collection, diagnostic referral, retrieval of results, and linking persons with active TB to care) minimised leakages in the diagnostic and treatment cascade, ensuring timely evaluation, confirmatory diagnosis, and treatment initiation of confirmed TB cases, resulting in a 97% treatment initiation rate. This is similar to initiation rates reported from other ICF initiatives.^14,15^ A retrospective records review in private healthcare facilities in Nigeria found a lower treatment initiation rate of 92%.^16^ A 25% cumulative treatment initiation rate at the point of seeking care initially was also reported for a group of high TB-burden countries. This reflects the challenges in treatment initiation where no active linkage to treatment is available, as in our intervention.^17^ The hub and spoke approach we deployed to help link people with presumptive TB to diagnostic facilities and treatment centres mirrors recommendations for a patient-centred approach to care. In this approach, mechanisms are put in place to facilitate access to appropriate diagnostic and treatment services for persons who are likely to have TB regardless of their initial level of contact with health services. ‘It enables access to quality services via patients’ preferred providers, at the level that is most accessible, affordable, and appropriate for them’.^17^

The higher TB diagnosed among persons aged 25–34 years and 35–44 years, and the lower TB among children less than 15 years in our study are similar to other findings in Nigeria.^18^ A 15-year retrospective study in Jos, Plateau State, which is one of our study sites, reported a TB prevalence of 54.8% and 48.8% among persons with and without HIV, respectively.^18^ This points to the contribution of other factors to the higher TB prevalence among this age group, such as smoking, alcohol consumption, poverty, and overcrowding.^19^ A study in Uganda found that the contributions of smoking, poverty, overcrowding, and alcohol use to TB prevalence were respectively 26.4%, 39.5%, 57.3% and 50.7%.^20^ Thus, addressing these wider determinants significantly helps control TB and other communicable and non-communicable diseases.

In Nigeria, adherence to regulations against selling, advertising, promoting, and sponsoring tobacco is still inadequate, and needs further enforcement.^21^ The situation is even worse for alcohol for which there is no dedicated regulation.^22^ The lower TB among children less than 15 years old despite our ICF intervention points to persistent problems with child TB detection. These include the non-specific nature of symptoms and signs of TB in children, the effect of the paucibacillary nature of childhood TB on TB smear positivity, and the challenge of obtaining good-quality sputum or gastric aspirate samples, especially in younger children.^23^ To improve TB case detection among children, initiatives such as children-focused health facilities and community-based ICF initiatives, increase in capacity of healthcare workers with high index of suspicion for TB, and increased funding for TB to scale up access to healthcare services are considered important as well as other recommended in WHO’s roadmap towards ending TB in children and adolescents would be important.^24^

As with other epidemiological findings, more men were diagnosed with TB than women in our study.^14^ This may be associated with the higher prevalence of major risk factors for TB among men compared to women, including smoking, alcohol consumption and incarceration.^25^ It may also be a result of lower sputum culture sensitivity among women;^26^ women may also show lower TB rates due to early screening as part of their healthcare seeking-related to pregnancy and childbirth.^27^ It is important, however, to ensure that particular attention is paid to addressing TB in women because of their peculiar vulnerabilities and impact on childhood TB.^28^ This could involve including maternal health services service delivery points in active TB case-finding initiatives at all levels of the health care system.

Our study could benefit from broader coverage of facilities across the entire TBLON 1&2 project. However, our report from more than 95% of the LGAs in each of the states in our study provides good representative data to support policy and programming decisions in the affected states and others with similar socio-demographic peculiarities and TB burden. Cost-effectiveness should be considered before scaling up ICF to low TB burden areas. Our study may also be affected by some degree of data completeness, as some omissions may have occurred due to manual data collection. The monthly data quality reviews instituted as part of the project may enable us to address this limitation, ensuring high-quality and complete data to monitor project performance. Aside from the 33% average contribution to TB notification rates, we could not compare the results of the ICF reported in this paper with outcomes from routine non-ICF settings. This was due to challenges accessing the requisite data from routine government programs in Nigeria. This gap should be addressed in future reports.

## CONCLUSION AND RECOMMENDATION

Facility-based ICF is an efficient strategy to increase TB notifications and address the challenge of missing TB cases in Nigeria’s efforts to end TB. Government and partners should continue promoting this strategy in public and private health facilities for optimal impact

## References

[1] Vos T, Global burden of 369 diseases and injuries in 204 countries and territories, 1990–2019: a systematic analysis for the Global Burden of Disease Study 2019. Lancet. 2020;396(10258):1204–1222. 33069326 10.1016/S0140-6736(20)30925-9 PMC7567026

[2] World Health Organization. Global tuberculosis report, 2023. Geneva, Switzerland: WHO, 2023.

[3] Stop TB Partnership. Global plan to end TB 2023–2030. Geneva, Switzerland: Stop TB Partnership, 2023.

[4] World Health Organization. Implementing the end TB strategy: the essentials. Geneva, Switzerland: WHO, 2015.

[5] Federal Ministry of Health. End-term review of Nigeria’s national strategic plan for tuberculosis control 2015-2020. Abuja, Nigeria: National Tuberculosis and Leprosy Control Program (NTBLCP), 2020.

[6] Otu A. A review of the national tuberculosis and leprosy control programme (NTBLCP) of Nigeria: challenges and prospects. Ann Trop Med Public Health. 2013;6(5):491.

[7] Stop TB Partnership. StopTB field guide 4: intensified TB case finding at facility level. Geneva, Switzerland: Stop TB Partnership, 2018.

[8] Federal Ministry of Health. National strategic plan for tuberculosis control 2021-2025. Abuja, Nigeria: National Tuberculosis and Leprosy Control Programme (NTBLCP), 2021.

[9] Fenta MD, Facilitators and barriers to tuberculosis active case findings in low- and middle-income countries: a systematic review of qualitative research. BMC Infect Dis. 2023;23(1):515. 37550614 10.1186/s12879-023-08502-7 PMC10405492

[10] United States Agency for International Development. USAID establishes two new $45 million ‘local organizations networks’ to fight TB in Nigeria. Washington DC, USA: USAID, 2020.

[11] World Health Organization. WHO consolidated guidelines on tuberculosis. Module 2: screening - systematic screening for tuberculosis disease. Geneva, Switzerland: WHO, 2021. 33822560

[12] The Global Fund. Best practices on TB case finding and treatment: reflections and lessons from West and Central Africa and beyond. Geneva, Switzerland: The Global Fund, 2018.

[13] Shah S, Intensified tuberculosis case finding among HIV-infected persons from a voluntary counseling and testing center in Addis Ababa, Ethiopia. J Acquir Immune Defic Syndr. 2009;50(5):537–545. 19223783 10.1097/QAI.0b013e318196761c

[14] Ogoamaka C, The TB surge intervention: an optimized approach to TB case-finding in Nigeria. Public Health Action. 2023;13(4):136–141. 38077724 10.5588/pha.23.0039 PMC10703131

[15] Eyo AS, A multi-faceted approach to tuberculosis active case finding among remote riverine communities in Southern Nigeria. Int J Environ Res Public Health. 2021;18(18):9850. 34574349 10.3390/ijerph18189424 PMC8472435

[16] Oladimeji O, Treatment outcomes of drug susceptible tuberculosis in private health facilities in Lagos, South-West Nigeria. PLoS One. 2021;16(1):e0244581. 33471851 10.1371/journal.pone.0244581 PMC7816975

[17] Hanson C, Finding the missing patients with tuberculosis: lessons learned from patient-pathway analyses in 5 countries. J Infect Dis. 2017;216(Suppl 7):S686–95. 29117351 10.1093/infdis/jix388 PMC5853970

[18] Sariem CN, Tuberculosis treatment outcomes: a fifteen-year retrospective study in Jos-North and Mangu, Plateau State, North - Central Nigeria. BMC Public Health. 2020;20(1):1224. 32781994 10.1186/s12889-020-09289-x PMC7422002

[19] Narasimhan P, Risk factors for tuberculosis. Pulm Med. 2013;2013:828939. 23476764 10.1155/2013/828939 PMC3583136

[20] Kirenga BJ, Tuberculosis risk factors among tuberculosis patients in Kampala, Uganda: implications for tuberculosis control. BMC Public Health. 2015;15:13. 25604986 10.1186/s12889-015-1376-3 PMC4311451

[21] Egbe CO, Bialous SA, Glantz S. Framework Convention on Tobacco Control Implementation in Nigeria: Lessons for Low- and Middle-Income Countries. Nicotine Tob Res. 2019;21(8):1122–1130. 29660032 10.1093/ntr/nty069 PMC6636173

[22] Abiona O, Oluwasanu M, Oladepo O. Analysis of alcohol policy in Nigeria: multi-sectoral action and the integration of the WHO ‘best-buy’ interventions. BMC Public Health. 2019;19(1):810. 31234812 10.1186/s12889-019-7139-9 PMC6591910

[23] Dodd PJ, Burden of childhood tuberculosis in 22 high-burden countries: a mathematical modelling study. Lancet Glob Health. 2014;2(8):e453–459. 25103518 10.1016/S2214-109X(14)70245-1

[24] Nkereuwem E, Kampmann B, Togun T. The need to prioritise childhood tuberculosis case detection. Lancet. 2021;397(10281):1248–1249. 33765409 10.1016/S0140-6736(21)00672-3 PMC9214637

[25] Marçôa R, Tuberculosis and gender - factors influencing the risk of tuberculosis among men and women by age group. Pulmonology. 2018;24(3):199–202. 29754721 10.1016/j.pulmoe.2018.03.004

[26] Diwan VK, Thorson A. Sex, gender, and tuberculosis. Lancet. 1999;353(9157):1000–1001. 10459926 10.1016/S0140-6736(99)01318-5

[27] World Health Organization. WHO recommendations on antenatal care for a positive pregnancy experience: screening, diagnosis and treatment of tuberculosis disease in pregnant women: evidence-to-action brief: highlights and key messages from the World Health Organization’s 2016 global recommendations. Geneva, Switzerland: WHO, 2022.

[28] McLaren ZM, Gender patterns of tuberculosis testing and disease in South Africa. Int J Tuberc Lung Dis. 2015;19(1):104–110. 25519799 10.5588/ijtld.14.0212

